# Using intervention mapping to develop an implementation strategy to improve timely uptake of streamlined birth-dose vaccines in the Democratic Republic of the Congo

**DOI:** 10.1371/journal.pgph.0002641

**Published:** 2024-01-25

**Authors:** Alix Boisson-Walsh, Bruce Fried, Christopher M. Shea, Patrick Ngimbi, Nana Mbonze, Martine Tabala, Melchior Mwandagalirwa Kashamuka, Pélagie Babakazo, Marcel Yotebieng, Peyton Thompson

**Affiliations:** 1 Department of Health Policy and Management, Gillings School of Global Public Health, The University of North Carolina, Chapel Hill, North Carolina, United States of America; 2 Ecole de Santé Publique de Kinshasa, Kinshasa, Democratic Republic of the Congo; 3 Division of General Internal Medicine, Department of Medicine, Albert Einstein College of Medicine, Bronx, New York, United States of America; 4 Division of Infectious Diseases, Department of Pediatrics, University of North Carolina, Chapel Hill, North Carolina, United States of America; University of Washington, UNITED STATES

## Abstract

Despite the policy recommendation and effectiveness of administering the hepatitis B birth-dose vaccine (HepB-BD) to newborns to prevent mother-to-child hepatitis B transmission, timely uptake remains an issue. Countries adopting the HepB-BD to their national immunization schedule report programmatic challenges to administering the vaccine within the recommended 24-hour window after delivery. Further, while the World Health Organization recommends streamlining three birth-dose vaccines (HepB-BD, BCG, and OPV0), scarce Sub-Saharan(SSA)-based literature reports on a streamlined and timely approach to birth-dose vaccines. As more SSA countries adopt the new birth-dose vaccine to their immunization schedules, a systematically developed implementation strategy—Vaccination of Newborns–Innovative Strategies to Hasten Birth-Dose vaccines’ delivery (VANISH-BD)—will facilitate the adoption and implementation of timely birth-dose vaccine uptake. In this paper, we describe the development of the implementation strategy using intervention mapping, an evidence-based and theory-driven approach. We report on the development of our intervention, beginning with the needs assessment based in Kinshasa Province, Democratic Republic of the Congo (DRC), informing step 1 of intervention mapping. The intervention is contextually relevant, locally produced, sustainable, and designed to improve timely birth-dose vaccine uptake in the DRC. We intend to inform future implementers about improving timely and streamlined birth-dose vaccine uptake and for VANISH-BD to be adapted for similar contexts.

## Introduction

Birth-dose vaccines, such as Bacillus Calmette- Guerin (BCG) and the first oral polio dose (OPV0), play a critical role in protecting infants from life-threatening diseases and prevent severe morbidity for millions of children every year [1–3]. Likewise, hepatitis B vaccine (HepB-BD) has been shown to dramatically reduce mother-to-child transmission of hepatitis B virus if administered within 24 hours of an infant’s birth (birth-dose), followed by the recommended subsequent doses [4]. This evidence led the World Health Organization (WHO) to recommend since 2009 that countries adopt the universal birth-dose vaccine, irrespective of maternal HBV serostatus [4, 5].

Despite the policy recommendation and effectiveness of administering timely HepB-BD to newborns to prevent mother-to-child hepatitis B transmission, timely HepB-BD implementation poses programmatic challenges. While 14 of 48 countries in sub-Saharan Africa (SSA) have introduced HepB-BD to their national immunization schedule, [6] timely uptake has remained strikingly low across the board. A 2022 study in Nigeria found that among the study sample, although 91% of infants received HepB-BD, only 33% received the vaccine within 24 hours of delivery [7]. A meta-analysis of 31 studies conducted in SSA found that the pooled rate of uptake of HepB-BD in the first 24 hours after delivery was 1.3% [8]. Even in high-income settings such as the United States, where HepB-BD was introduced in 1991, the complex challenge of timely delivery of HepB-BD led to high rates of delayed administration. A 2020 New York state study testing an intervention to improve timely administration through strategies such as staff education and guideline enforcement improved timely uptake from 40% to 92% of infants receiving HepB-BD [9]. As more S SA countries adopt the new birth-dose vaccine to their immunization schedules, the unique challenges demonstrated by these prior studies illustrate that implementation strategies are needed to promote uptake of new birth dose vaccines in a new context. A systematically developed implementation strategy will facilitate the adoption and implementation of timely birth-dose vaccine uptake.

We used intervention mapping (IM) to develop an evidence-based and theory-driven implementation strategy to facilitate the streamlined uptake of timely birth-dose vaccines, further described below. Here we document the development of VANISH-BD (Vaccination of Newborns–Innovative Strategies to Hasten Birth-Dose vaccines’ delivery), an implementation strategy to streamline birth-dose vaccine delivery in a timely manner using an IM approach. While we tailored the intervention development process to a DRC setting, our objective is to evaluate the implementation strategy’s feasibility so that other researchers may adapt VANISH-BD to improve timely birth-dose vaccine uptake in other settings.

## Methods

### Setting

The intervention mapping process was set in Kinshasa Province, the DRC, a vast area with an estimated 15 million inhabitants [10]. The province encompasses urban, peri-urban, and rural areas, is one of the world’s fastest-growing megacities, and is a contender as the most populous metropolitan area in Africa. Kinshasa Province is divided into 35 health zones with one reference hospital each; the number of facilities varies based on the size of the health zone [11]. The predominant facility ownerships in the DRC are religious, private, for-profit, and public [11]. In the DRC, the national vaccine program (Programme Élargi de Vaccination; PEV) selects facilities to provide vaccine services based on quality, and the number of individuals served rather than channeling vaccines only through publicly-owned facilities. For example, PEV may select a religious-based facility that reaches ample constituents to provide vaccination despite it not being government operated. Currently, PEV recommends two vaccines at birth, BCG and OPV0. OPV0 is followed by additional doses at 6, 10, and 14 weeks of age, which are the same time points at which the pentavalent vaccine (containing HepB3) is recommended. The final vaccine-related setting consideration is that community health workers, employed at the health zone level, conduct vaccine outreach to improve uptake.

This study reports on the process of developing a theory-driven implementation strategy to improve the timely uptake of birth-dose vaccines in Kinshasa Province by using preliminary findings from this particular setting. A future study will evaluate the feasibility of VANISH-BD so that it can be scaled up or adapted by implementers in other similar settings.

### Open forum recruitment

As part of Step 2, Performance and change objectives, we conducted an open forum with health staff at both the health zone and facility levels. From August to September 2022, the open forum participants were recruited from study facilities and health zones, specifically selected based on their prior involvement in the needs assessment phase of our study [12].

### Ethics statement

Informants provided written, informed consent to participate in the needs assessment and the open forum. Participants were anonymized using unique identification numbers, ensuring confidentiality throughout the process and all identifying information was securely stored on a dedicated server. Institutional Review Boards at UNC-Chapel Hill and the Kinshasa School of Public Health approved the study protocol [UNC IRB 21–0014; KSPH IRB 00011-04101-00001365292-20].

### Intervention mapping

Intervention mapping is a systematic approach that outlines a six-step process, using evidence and theory to introduce interventions and develop related implementation strategies for addressing health problems. Researchers and implementers have used intervention mapping in studies globally—and in sub-Saharan Africa specifically—to develop implementation strategies for introducing health interventions ranging from cancer management, [13] malaria elimination, [14] and improving antenatal care [15]. However, to our knowledge, studies have yet to employ an intervention mapping approach to develop an implementation strategy to improve the timely uptake of streamlined birth-dose vaccines.

IM is an iterative and cumulative process that uses outputs from previous steps to inform subsequent steps in the implementation process. The six steps of the IM process include:

1. A needs assessment and logic model of the problem,
2. Development of performance and change objectives,
3. Selection of theory-based methods and strategies,
4. Development of program components and materials,
5. Development of the implementation and adoption plan, and
6. Development of the evaluation plan.

Step 1 begins with an initial needs assessment focusing on the analysis of existing factors–both barriers and facilitators–related to the health problem. The analysis guides the development of a logic model of the problem. Step 2 integrates the findings from the needs assessment to select a target population and target performance and change objectives. Step 3 requires selecting theory-based methods and practical strategies used in step 4 to design the intervention program and materials. Step 5 concentrates on program adoption and implementation, and step 6 generates the instruments to evaluate the intervention [16].

An advantage of the IM process is its grounding in conceptual models, behavioral theory, and engagement with key stakeholders and the community members to develop context-appropriate interventions. Community-based participatory research (CBPR) is an approach that ensures the use of bi-directional learning efforts to understand and improve intervention strategies [17]. CBPR provides a link through which evidence-based research, community members, and leaders can combine to develop sustainable and context-specific interventions. IM emphasizes applying CBPR principles by engaging community stakeholders throughout the intervention’s development, implementation, and evaluation.

## Results

### Step 1. Needs assessment

In this step, we conducted formative research to define the priority population and environmental change agents using a literature review and qualitative and quantitative research approaches. All three of the components of the needs assessment are previously published analyses [12, 18, 19] that are foundational to the intervention mapping approach.

#### Literature review

Our assessment began with a literature review of barriers and facilitators to the timely uptake of HepB-BD in sub-Saharan Africa to review lessons learned from other similar contexts [18]. The review consisted of 49 articles and highlighted the need for a multi-level initiative to target factors impacting timely birth-dose vaccine uptake at the policy, facility, and community levels. The review also identified research supporting streamlined efforts of HepB-BD, polio, and BCG birth dose vaccines to improve timely uptake. At the time of the review’s publication, four SSA-based studies assessed the effect of streamlined vaccines outside the 24-hour window. However, no study had evaluated the effect of streamlining the three vaccines at birth [8]. The WHO confirms that the three vaccines do not interfere with one another’s immune response, and should be administered at birth [20]. Therefore, timely, streamlined birth-dose vaccines are a proven approach to improving infant health outcomes.

#### Qualitative research

We conducted interviews with expectant mothers and informants at the health facility, health zone, and national level (n = 30) across Kinshasa Province [12]. These interviews explored determinants to the uptake of currently available birth-dose vaccines–BCG and OPV0 –and perceived barriers to future uptake of HepB-BD vaccine from various perspectives. We conducted the interviews in seven health facilities, two health zone administration offices, and the national PEV offices. Facility staff working the day of and mothers visiting the center for a prenatal visit were approached and recruited for the study. Findings from this study highlighted significant barriers to the uptake of birth dose vaccines, including regular stockouts and lack of storage capacity, inconsistent vaccine fees across facilities, lack of communication between delivery and vaccine departments, and limited understanding of vaccines among mothers and communities [12].

#### Quantitative research

We conducted regression analyses leveraging longitudinal data from a continuous quality improvement study (NCT03048669) to assess the barriers to the timely administration of currently available birth-dose vaccines, BCG and OPV0 [19]. We also evaluated the first dose of the pentavalent vaccine (including HBV) at six weeks. The study sample spanned 105 health clinics. From the total study sample of 2,800 women, the sub-study included 2,398 eligible mother-infant dyads. The sub-study exclusion criteria included missing data about the infant’s vaccine uptake, death of the mother or infant, and loss of follow-up before the study team could capture vaccination information. The longitudinal data analyzed the impact of factors such as a mother’s socio-demographics and a health facility’s general readiness score on an infant’s timely receipt of vaccines. At the individual level, results showed that a mother’s education, wealth, and proximity to a facility influenced the timely uptake of vaccines. A mother visiting a facility with religious affiliation and high general and immunization-specific readiness also impacted the timely uptake of vaccines.

#### Conceptual model

The PRECEDE (Predisposing, Reinforcing, and Enabling Constructs in Educational/Environmental Diagnosis and Evaluation) model guided our first step of IM. PRECEDE is a comprehensive structure often used in IM research to evaluate health needs to guide the design and implementation of health interventions [21]. PRECEDE categorizes factors influencing the health problem using three levels: individual (predisposing), interpersonal (enabling), and structural/ policy (reinforcing) factors inherent in health behaviors and interventions. Findings from the three parts of the needs assessment were categorized using the PRECEDE model in Table 1.

**Table 1:** Preliminary findings from need assessment.

PRECEDE Construct	Preliminary Findings		Source
Individual (Predisposing)	Facilitators	Motivation to keep infant safe and protected	Qual
		More vaccine knowledge with more mature age and education	Qual & Quant
		Knowledge that vaccines can protect against seasonal diseases	Qual
	Barriers	Low socioeconomic status of mother	Quant
		Lack of knowledge about disease prevalence and vaccine benefits	Qual
		Delay seeking birth-dose vaccines	Quant
		Residing further than walking distance from a facility	Quant
Interpersonal (Reinforcing)	Facilitators	Opportunity exists to disseminate vaccine knowledge during ANC visits among mothers who seek antenatal care	Qual
		Opportunity exists to disseminate vaccine education to community by leveraging CHWs	Qual
		Physical space for posters and guidance in facilities	Qual
	Barriers	Collaboration and communication between midwives and vaccine staff	Qual
		Influence on vaccine hesitancy from families and friends (community)	Qual
		Clarity of vaccine education disseminated to mothers during ANC visits	Qual
Organizational (Enabling)	Facilitators	Existing guidelines and workflows in place for vaccine distribution	Qual & Quant
		Free vaccine services at public facilities	Qual
		Discussions at national level to include HepB-BD to national immunization schedule	Qual
	Barriers	Absence of guidelines about streamlining timely birth-dose vaccines across facilities	Qual & Quant
		Varying vaccine fees across facilities	Qual
		Stockouts of vaccines	Qual
		Absence of storage for birth-dose vaccines in delivery ward	Qual
		Absence of HepB-BD on national immunization schedule	Qual & Quant

### Step 2. Performance and change objectives

In the second step, we employed the main findings from the needs assessment to define birth-dose vaccine uptake behaviors for facility staff and mothers, defined our study objectives, described determinants of the identified behaviors, and developed matrices of change.

Our ultimate objective was to share findings with the national government to support modifying the national immunization policy and administration guidelines surrounding newborn immunization in Kinshasa Province, the DRC. Therefore, in this step, we aimed to develop, implement, and examine the feasibility of an intervention streamlining birth-dose vaccines, including HepB-BD, BCG, and OPV0, within 24 hours of delivery for newborns in Kinshasa. We achieved our objective by using a two-pronged approach targeting facilities and leveraging community outreach.

Change matrices help researchers to determine the factors that must change to bring about each performance objective [22]. Using the findings from the needs assessment, we produced change matrices that combined factors likely to be associated with the achievement of our listed objectives. Our change matrices included both performance objectives and the determinants of achieving each objective. We divided the matrices into two target groups, the adopters–decision-makers at the health zone and facility levels, and the implementers–health staff at the facility and community level (S1 Table). Determinants included beliefs, behaviors, and expectations specific to each role, i.e., adopters and implementers. Any barrier determined through our needs assessment was therefore transformed into a change objective to achieve our desired outcome [14, 22].

As an illustrative example within our intervention strategy, consider Performance Objective 2, targeting our adopters, the health zone and facility decision-makers and leaders. This objective centers on their agreement to expand vaccine services to include HepB-BD. To achieve this, they need to acquire specific knowledge, encompassing (a) the ability to communicate the unmet vaccine need among infants and (b) a comprehensive understanding of the steps necessary to expand birth-dose vaccine services. Concurrently, building essential skills and self-efficacy involves fostering confidence in (a) their capacity to collaborate with partners and decision-makers to extend BD vaccine services and (b) their facility’s capability to adapt workflows to incorporate the intervention. The anticipated outcome is that expanded BD vaccine services will ultimately reduce infant morbidity and mortality rates. Furthermore, normative beliefs to promote this transformation include instilling the belief that other leaders and facilities are actively endorsing the expansion of BD vaccine services.

Another example, applicable to all health staff implementers, involves Performance Objective 1 centered around program implementation and participation in a comprehensive two-day study training program. The training serves as a platform for describing (a) the study components in a user-friendly and implementable manner and (b) the processes for effectively utilizing guidelines and materials. Skill development lies in the demonstrated confidence of health staff in their ability to attend the training and absorb its content effectively. Their expected outcomes encompass an enhanced readiness to successfully implement the intervention and receive recognition from program champions for completing the training. It is critical to foster the normative belief that attending the training is a collective effort, with the expectation that their colleagues will also actively participate and benefit from the training’s insights and skills development.

To define the change objectives, we reported outcomes from needs assessment findings to community members. We held an open forum with health staff at the health zone and facility levels to elicit feedback and discuss reasonable change objectives. Each of the three sessions included approximately five participants and five study staff. Among the participants were a facility director, heads of vaccination, antenatal and maternity divisions, and a health zone representative. Sessions included dissemination of findings from the needs assessment, accompanied by an informative handout highlighting the critical determinants to timely birth-dose vaccine uptake. We then held group discussions to inform decisions on desired project outcomes for behavior change.

### Step 3. Select theory-based methods and strategies

The objective of step 3 was to identify specific strategies to improve the determinants of success described in step 2. We therefore identified theoretical concepts and practical applications to guide the intervention’s implementation. We identified methods for changing determinants from those outlined in step 2 and selected delivery strategies (S2 Table). The community and health staff required sustained behavior change to improve timely birth-dose vaccine uptake. Therefore, we chose two relevant theories, the Theory of Planned Behavior [23] and the Social Cognitive Theory, [24, 25] both widely employed to predict and explain health behavior in health-related interventions. The Theory of Planned Behavior states that behavior depends on an individual’s motivation and ability to control behavior. The theory is categorized into three types of beliefs–behavioral, normative, and control [23]. The SCT suggests that bidirectional interaction between an individual and their behavior and environment leads to behavior change. The key components which impact behavior highlighted by the SCT include self-efficacy, behavioral capability, expectations, self-control, observational learning, and reinforcements [24, 25]. Therefore, while we used the SCT to frame the macro-level factors influencing timely birth-dose vaccine uptake, the Theory of Planned Behavior focused on the micro-level components influencing the individual’s health behavior. Combining the two theories, we could predict and explain health behavior at various levels.

Using both theories, we successfully organized determinants of behavior change and theory-based methods with practical application of behavior change techniques to overcome targeted barriers (S2 Table). Using the theoretical frameworks, we grouped our strategies into three levels to impact sustained behavioral change: increasing knowledge, increasing self-efficacy, and changing outcome expectations. We employed the theory-based methods and linked each to practical application within the intervention’s context. As an illustration, within the framework of enhancing skills and self-efficacy, we incorporate theory-based change methods, such as skill building and guided practice, reinforcement, monitoring and feedback, and environmental restructuring. Each method entails specific activities essential for the actors involved to achieve their goals.

For instance, in environmental restructuring, study implementers are tasked with modifying vaccine fees to ensure they are affordable or entirely free, establishing consistency across all facilities. Facility-based decision-makers play a pivotal role by seamlessly integrating guidelines and materials into their workflow, prompting facility staff to apply these guidelines to every mother-infant pair they serve consistently. As part of this process, all facility staff members convene to review and familiarize themselves with the new guidelines and protocols, facilitated by elected champions who help disseminate and ensure adherence to these new procedures. Finally, the vaccine staff’s contribution is integral, as they provide transportation vouchers to mothers who rely on public transportation to attend vaccine appointments, ensuring equitable access to healthcare services. It is essential to emphasize that these activities are indispensable to achieving effective environmental restructuring, demonstrating the interconnectedness and collaborative effort required to bring about meaningful change.

**Fig 1:**
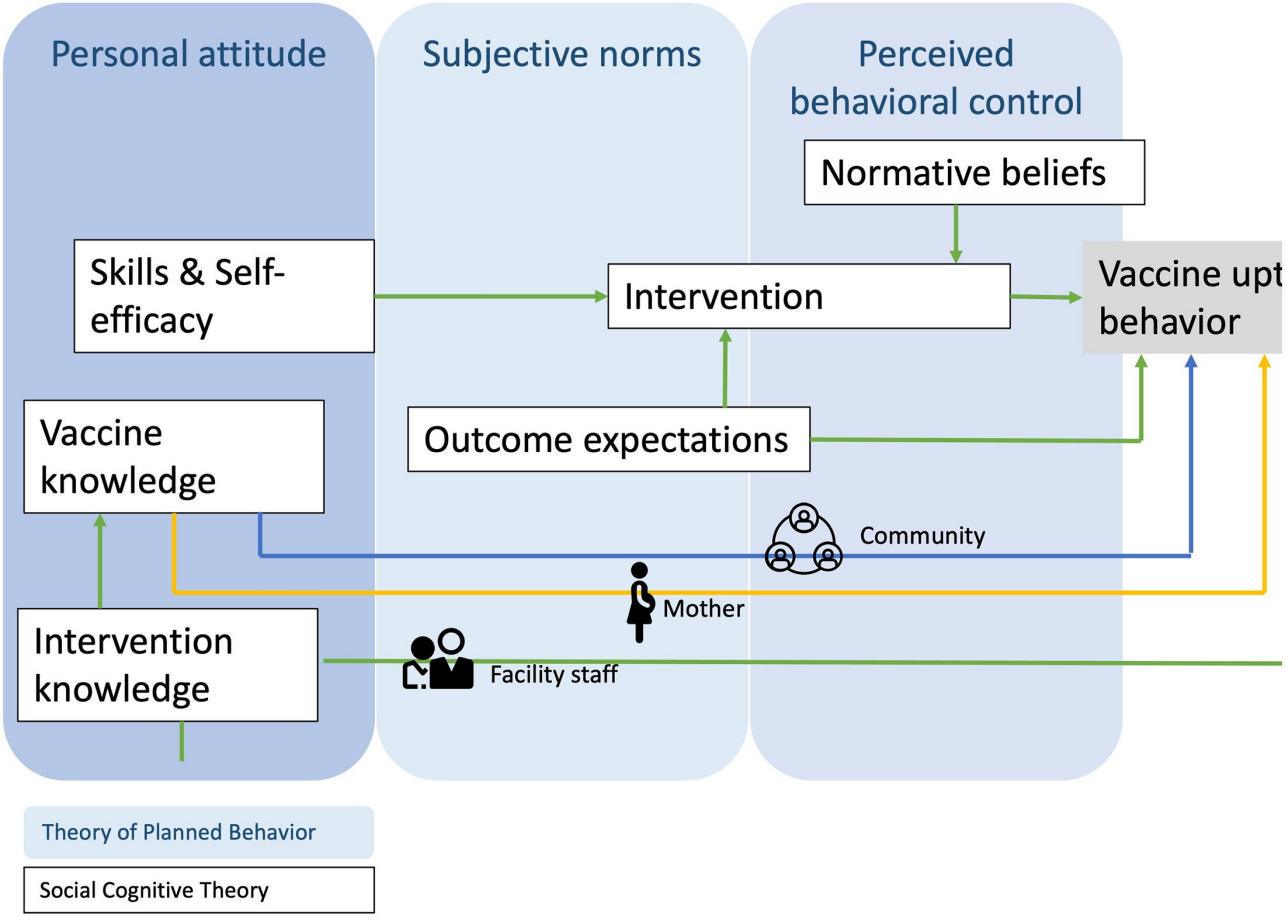
Theory-driven logic of solution model for vaccine uptake.

Translating methods into applications required a sufficient understanding of the theory behind the method, leading to the theoretical change process. We constructed a logic model illustrating the potential relationships between theory and evidence-based methods and how they influenced determinants and outcomes (Fig 1). This model underpinned the intervention’s planning, implementation, and evaluation.

### Step 4. Develop the intervention

The next task for developing the intervention was to design educational and training materials and associated protocols for use by other policymakers and implementers aspiring to improve the uptake of timely and streamlined birth-dose vaccines. We assessed the existing resources at the facility and health zone levels, and examined how those resources, modified versions of the resources, or new assets could address our named program objectives. Based on the findings from the needs assessment, two program components were deemed important. First, a need to focus on behavioral aspects through individual and community awareness. Second, more buy-in from and guidance at the facility level are necessary to sustain a streamlined birth-dose vaccine approach. We employed implementation strategies to address the two components, including developing adopter and implementer relationships, identification of intervention champions, training sessions, and modification of educational materials, guidelines, and protocols (VANISH-BD). The two overarching components–updating material for individual and community awareness and training champions to ensure sustainability–are discussed below.

#### Updated material for individual and community awareness

Other relevant research and the needs assessment informed VANISH-BD’s components for achieving specified program objectives. Our assessment found that mothers received little to no information about childhood vaccines during ANC visits, a missed opportunity to disseminate vaccine knowledge. In addition, the information shared varied across facilities, with no clear guidance on timely birth-dose vaccine uptake. Therefore, we developed new educational posters and scripts to be used by ANC staff to educate mothers about the risks of hepatitis B, polio, and tuberculosis and the benefits of the vaccines to prevent these diseases. In addition, our research suggested that the community plays a significant role in a mother’s decision to vaccinate her infant. Therefore, we sought to leverage CHWs to partner with community leaders–through churches and schools–to further disseminate information about newborn vaccines. We developed specific guidelines and educational materials for this purpose. Finally, we found the following: only vaccine ward staff were administering the birth-dose vaccines, current guidelines did not ensure that delivery and vaccine ward staff communicate when newborns are born, and fridges in the maternity ward do not stock birth-dose vaccines. Each of these factors inhibited the timely uptake of streamlined birth-dose vaccines. Therefore, we developed updated protocols for birth-dose vaccine administration and a checklist outlining the guidelines for timely vaccine uptake to be administered by maternity ward staff for each newborn. All materials were translated into local languages and dialects and used pictures when possible to reach illiterate audiences.

#### Training champions to ensure a sustained initiative

In order to introduce a feasible and sustainable implementation strategy, our suggestion was to train community health workers and staff in the vaccine ward and delivery wards as champions to implement VANISH-BD and ensure that other health staff adhere to the initiative. The use of champions served three purposes: (1) to ensure day-to-day activities are maintained, (2) to hold recurring meetings with champions for continuous learning, and (3) to provide training to staff and incite buy-in to ensure the sustainability of VANISH-BD. Available evidence suggests that champions are facilitators of successful change efforts and are crucial to practical implementation in health care [26]. Training, empowering, and leveraging champions is commonplace when implementing programs across global health systems. However, to date, most published research leveraging change champions is based on western research with scarce LMIC-based literature available.

The intervention planning group developed a two-day baseline training for champions and an additional two-days led by champions for relevant health staff. The training, which we will roll out during the trial phase, will consist of the study staff attending two days of onsite practice. The onsite practice will involve study staff visiting the facilities and supervising the implementation of VANISH-BD.

In the future, every month during the study, the three levels of champions will then meet with study staff to debrief about challenges encountered and brainstorm potential solutions going forward. The champions will play a critical role during the study to help flesh out issues related to the implementation strategy. This step will ensure that the final implementation package, VANISH-BD, and associated training and education material and guidelines are field-tested, and feasibility verified for scale-up or use in other settings.

### Step 5. Develop the implementation plan

Step 5 focused on developing an implementation plan to ensure the successful adoption and implementation of VANISH-BD across all study facilities. The objective was to implement the complete intervention in all study facilities and achieve sustainability through the maintenance of champions and training. From this study’s formative needs assessment step, and in response to the need for timely and streamlined birth-dose vaccines, we produced VANISH-BD’s intervention plan and materials for all of these strategies (Table 2). The intervention plan has been discussed, and the adoption and implementation of VANISH-BD assured by leadership at the health zone and facility levels.

**Table 2:** Specific implementation plans and materials for streamlining timely birth-dose vaccines.

Implementation Strategy	Implementation Plan
Facility based education	• Development and dissemination of single-page infographic about HepB-BD for use during antenatal visits and at delivery by health staff to educate mothers and families about HBV risk and HepB-BD benefit.
	• Development and dissemination of educational posters about HepB-BD and other BD vaccines to be hung in facilities
Educational outreach program	• Creation of a community outreach initiative in schools and churches
	• Leverage CHWs to educate the community on HBV and other vaccine preventable diseases (tuberculosis and polio)
Champion approach	• Training of champions in the vaccine ward, the delivery ward, and community health workers
	• Champion supervision at the onset of the study
Champion learning collaborative	• Monthly joint meetings via a WhatsApp platform of each champion team to share experiences with the study and discuss challenges and solutions.
Written clinical guidelines	• Dissemination across all study facilities of written guidelines for the introduction of HepB-BD alongside streamlined OPV0 and BCG

### Step 6. Develop the evaluation plan

The final step was developing an evaluation plan, which ensured intervention feasibility measurement spanning the lifecycle of its implementation. During the future study, a continuous evaluation will allow opportunities to amend the intervention and improve its adoption, impact, and sustainability. The evaluation plan will be finalized as part of the completed future study, but we present an overview below.

#### Study design and timing

We will evaluate the feasibility of VANISH-BD through a cluster-randomized controlled trial in Kinshasa Province, DRC, in 12 facilities. We will expose the treatment arm to VANISH-BD, and the control arm will receive the HepB-BD vaccine without the implementation package. The treatment and control facilities will be located in non-contingent catchment areas to ensure community dissemination does not spill over to the control facility territory [27]. We will organize an awareness session to inform the facility staff in the control arm that newborns will receive HepB-BD. We will divide the study period into two phases: a 12-month intervention and a three-month post-intervention follow-up phase. The 12-month intervention period of VANISH-BD will provide enough time for staggered facility roll-out, follow-up of enrolled mother-infant dyads, and time to deliver and follow the EPI schedule through the required 14 weeks. We will use the three-month post-intervention phase to clean and analyze study findings.

#### Outcomes

The primary outcome will be the proportion of newborns in facilities receiving timely birth-dose vaccines (within 24 hours of delivery) of all three vaccines, HepB-BD, BCG, and OPV0. The secondary outcome will be the number of children receiving a complete EPI schedule by 14 weeks of age, indicating a comprehensive shift in health behavior and awareness about the importance of vaccines. Both outcomes will be measured using birth and vaccine records at the facility and health zone levels. Health zone CHWs will help track down mother-infant dyads that leave or move from study facilities before completing their EPI schedule. A final outcome will be vaccine awareness and perceived benefit among health staff and mothers in the treatment and control groups. Awareness and perceived benefit will be measured using baseline and end-line questionnaires. The study staff will conduct a quarterly review of VANISH-BD’s implementation and will make necessary adjustments to the study’s approach.

## Discussion

In this paper, we presented an intervention mapping approach to develop VANISH-BD, a strategy targeting health staff to equip them with the necessary tools to increase the uptake of timely birth-dose vaccines for newborns. VANISH-BD addresses the barriers to vaccine uptake using theory-based behavior change techniques to achieve the desired behavior outcome in health staff, mothers, and the community. In the future, we will conduct a randomized controlled trial to assess the feasibility of VANISH-BD. While this initiative was developed specific to the DRC, we intend for VANISH-BD to be adapted and used in other contexts.

Our initiative was based on formative research to define the priority population and environmental change agents using a literature review as well as qualitative and quantitative research. We categorized our assessment using the PRECEDE model to understand the individual, interpersonal, and environmental components that impact health behavior influencing the uptake of birth-dose vaccines. The needs assessment highlighted significant barriers at the individual level, such as lack of vaccine knowledge among mothers and health staff; at the interpersonal level, such as a lack of communication between EPI and maternity staff; and at the environmental level, such as an absence of consistent guidance about birth-dose vaccines across facilities. Among our most significant challenges was the scarce literature on implementation strategies for effective rollout of timely HepB-BD in similar SSA settings. This gap in the literature pushed us to employ the IM approach to develop this initiative based on a needs assessment conducted in the DRC and to report on the findings so that other researchers and implementers may use the implementation strategy, VANISH-BD, in other vaccine uptake research.

Through our decision to use an IM approach that is grounded in theory and evidence, we can proactively address multilevel barriers to timely streamlined BD vaccine uptake. As such, our intervention focuses on updating educational materials to increase individual and community awareness as well as training champions to foster internal ownership and leadership of VANISH-BD and, ultimately, its sustainability. Maternal education has been shown to positively contribute to child health and associated immunization service uptake [28, 29]. A meta-analysis found that education about vaccinations through counseling sessions and printable information materials increased overall vaccination coverage by 19% [30]. In the same meta-analysis, interventions for providers, such as training for health staff and reminders for end-users, were shown to improve vaccine coverage by 13% [30]. Finally, leveraging CHWs to engage and educate community and religious leaders is a commonly employed approach to increase vaccine uptake through information campaigns [31–33]. Given the unique challenge of administering the HepB-BD within the short window after birth, an intervention leveraging all of these evidence-based approaches has the best likelihood of increasing the uptake of timely birth-dose vaccines.

Our study’s primary limitation is that there are external factors beyond the scope of what can be done at the facility and community levels to fully implement the VANISH-BD study. For instance, factors like stockouts of vaccines, which can significantly affect the implementation process, are beyond our control as their management is at the national and multilateral/international/donor levels. These external factors may impact the availability and accessibility of vaccines, potentially hindering the seamless execution of our implementation strategy.

An additional limitation is that our research operates within a broader context where scarce research exists on the novel approach of streamlining birth-dose vaccines within 24 hours of delivery. Our literature review revealed that, despite WHO’s recommendation of streamlined, timely birth-dose vaccines, research applying this approach had yet to be conducted in SSA. However, this gap in the literature propelled us to develop VANISH-BD, an evidence-based implementation strategy to improve the uptake of timely BD vaccines. While our approach has been tailored to our specific study setting, it may not be directly applicable in all contexts due to variations in healthcare systems and infrastructures. Nevertheless, our hope is that future researchers and implementers may draw insights from our evidence-based approach and adapt it to suit the unique circumstances of their respective settings.

## Conclusions

This study reports on developing VANISH-BD, an implementation strategy to improve the uptake of streamlined birth-dose vaccines within 24 hours of delivery. We developed VANISH-BD using an intervention mapping approach to ensure that it was grounded in theory. The intervention is contextually relevant, locally produced, sustainable, and designed to improve timely birth-dose vaccine uptake in the DRC. By providing a detailed, stepwise description of the intervention development process, other researchers and implementers may use the findings to adopt the implementation strategy in other relevant settings to facilitate HepB-BD introduction and improve the uptake of streamlined birth-dose vaccines at the facility level.

## Supporting information

S1 Table Matrices of change. (DOCX)

S2 Table Determinants, theoretical-based methods, and practical application for each study actor. (DOCX)

S1 Text Inclusivity in global research checklist. (DOCX)
