# Supplementary material for: Using intervention mapping to develop an implementation strategy to improve timely uptake of streamlined birth-dose vaccines in the Democratic Republic of the Congo
Source: PLOS Glob Public Health. 2024 Jan 25;4(1):e0002641. doi: 10.1371/journal.pgph.0002641 (PMC10810515; doi:10.1371/journal.pgph.0002641)
Supplement: S1 Table — (DOCX) [file pgph.0002641.s001.docx]

# S1 TABLE. MATRICES OF CHANGE

# MATRIX OF CHANGE FOR ADOPTERS

| **Target : role** |  | **Determinants** | | | |
| --- | --- | --- | --- | --- | --- |
|  | **Performance objectives** | **Knowledge** | **Skills & Self-efficacy** | **Outcome expectations** | **Normative beliefs** |
| Health zone & facility decision-maker and leaders : adopter | PO1. Agree to participate in the study | K1.a. Describe the components of the study | SSE1.a. Expresses confidence in the ability to do what is expected of the study | OE.1.a. Expect that the study intervention development partners will provide support with program implementation and resources | NB.1. Express belief that other facilities like theirs are agreeing to implement the study |
|  |  | K1.b. Describe the rates of timely birthdose vaccine uptake and HBV prevalence as problems that need to be addressed | SSE1.b. Perceives that the facility is capable of change | OE1.b. Expect this program will provide streamlined/timely birth-dose vaccine uptake |  |
|  |  |  | SSE1.c. Describes the facility as ready and able for change |  |  |
|  | PO2. Agree to expand vaccine services to include HepB-BD | K2.a. Express the unmet vaccine need among infants | SSE2.a. Express confidence in ability to work with partners and decision-makers to expand birth-dose vaccine service. | OE2.a. Expect that expanded birth-dose vaccine services will decrease infant morbidity and mortality rates | NB2.a. Express belief that other facilities are agreeing to expand birth-dose vaccine services |
|  |  | K2.b. Describe the steps needed to expand birth-dose vaccine services | SSE2.b. Express confidence in facility's ability to arrange workflow to incorporate study |  | NB2.b. Express belief that leaders and decision-makers will encourage expansion |
|  | PO3. Agree to participate in evaluation | K3.a. Describe the expected outcomes of the study | SSE3.a. Express confidence in ability to create records for evaluation | OE3.a. Expect the evaluation will add value to facility reporting | NB3. Express belief that other facilities in the study will participate in the evaluation |
|  |  | K3.b. Describe the procedures for participating in the evaluation | SSE3.b. Believe facility is a learning environment | OE3.b. Expect that evaluation results will add value and status as compared to other competing facilities |  |
|  |  |  |  | OE3.c. Believe evaluation results will help facility support program and garner future funding |  |
|  | PO4. Provide a program champion for the study | K4. Explain the responsibilities of the program champions in study | SSE4. Express confidence in the ability to recruit a program champion from each sub-group (midwives, vaccine staff, CHW) | OE4. Expect the program champion will enable the study to be implemented and maintained | NB4. Lists other clinics like theirs that use champions to assist in practice change |
|  | PO5. Assure procedures are in place for study implementation | K5. Describe process for ensuring study implementation procedures | SSE5. Demonstrate administrative ability to facilitate study implementation procedures | OE5. Expect that workflow procedures will improve staff engagement and efficiency | NB5. Express belief that other facilities in the study are also following implementation procedures |
|  | PO6. Oversee that procedures remain in place for sustained study implementation and workflow procedures | K6. Describe steps to assure sustained study implementation workflow and procedures | SSE.6. Demonstrate administrative ability to maintain ongoing program implementation | OE6. Expect that sustained workflow procedures will improve sustained study implementation | NB6. Express belief that sustaining the study is good for the facility |

# MATRIX OF CHANGE FOR IMPLEMENTERS

| **Target : role** |  | **Determinants** | | | |
| --- | --- | --- | --- | --- | --- |
|  | **Performance objectives** | **Knowledge** | **Skills & Self-efficacy** | **Outcome expectations** | **Normative beliefs** |
| Health staff : implementer   *All* | PO1. Facility staff agree to implement the program and attend 2-day study training. | K1.a. Describe the components of the study as easy to use and implement | SSE1. Demonstrate confidence in ability to attend and learn from training | OE1.a. Expect participating in training will ensure readiness to successfully implement the program | NB1. Expresses belief that their colleagues and other facility staff will attend trainings |
|  |  | K1.b. Describe process for using guidelines and materials |  | OE1.b. Expect champion and facility leadership will praise/acknowledge them for completing the training successfully |  |
| *Midwife : implementer* | PO2. Educates mothers about disease risk and vaccine benefits during ANC visits | K2. Awareness of study implementation procedures, guidelines, and workflows | SSE2.a. Demonstrate ability to deliver and maintain use of education material | OE2.b. Expect to make a difference and disseminate knowledge | NB2. Express belief that educating mothers makes a difference in infant morbidity and mortality |
|  |  |  | SSE2.b. Demonstrate ability to adhere to study workflow procedures | OE2.b. Expect that workflows and procedures will aid study implementation |  |
|  | PO3. Coordinates with vaccine staff | K3. Awareness of study implementation procedures, guidelines, and workflows | SSE3.a. Demonstrate ability to coordinate vaccine administration | OE3.a. Expect that coordination between vaccine and delivery staff will improve timely birth-dose uptake | NB3. Express belief that midwives taking on additional step of coordinating vaccines for newborns will increase timely birth-dose vaccine uptake |
|  |  |  | SSE3.b. Demonstrate ability to adhere to study workflow procedures | OE3.b. Expect that workflows and procedures will aid study implementation |  |
|  | PO4. Uses barrier scripts to respond to mother concerns about vaccination | K4. Awareness of study implementation procedures, guidelines, and workflows | SSE4.a. Express confidence using strategies to increase vaccine uptake | OE4.a. Expect to make a difference and disseminate knowledge | NB4. Express belief that educating mothers makes a difference in avoidable infant morbidity and mortality |
|  |  |  | SSE4.b. Demonstrate ability to adhere to study workflow procedures | OE4.b. Expect that workflows and procedures will aid study implementation |  |
| *Vaccine staff : implementer* | PO5. Captures infants' immunization status | K5. Awareness of study implementation procedures, guidelines, and workflows | SSE5.a. Demonstrate ability to regularly capture vaccine status at the end of every day | OE5.a. Expect to improve facility and study tracking of infants' vaccine information | NB5. Express belief that their role capturing infant vaccine status every day improves vaccine tracking and monitoring |
|  |  |  | SSE5.b. Demonstrate ability to adhere to study workflow procedures | OE5.b. Expect that workflows and procedures will aid study implementation |  |
| *Community health worker : implementer* | PO6. Partner with community leaders to improve preventable disease and vaccine awareness in community | K6. Awareness of study implementation procedures, guidelines, and workflows | SSE6.a. Demonstrate ability to recruit willing community leaders | OE6. Expect that community awareness of vaccines will improve over time through community-leadership involvement | NB6. Expresses belief that they are able to recruit community leaders to support VANISH-BD |
|  |  |  | SSE6.b. Demonstrate ability to disseminate vaccine education to community leaders |  | NB6.b. Expresses belief that involving community leaders will make a difference in avoidable infant morbidity and mortality |
| *Champion: implementer* | PO7. Champions oversee implementation efforts and provide feedback to facility staff | K7.a. Awareness of daily and weekly activities associated with champion's role | SSE7. Demonstrates confidence and ability to oversee implementation of the study | OE7. Expect that through continuous monitoring and communication, the study will be implemented effectively | NB7. Believes that other champions in other clinics are conducting the same role |
|  |  | K7.b. Awareness of steps needed to monitor implementation |  |  |  |
|  | PO8. Champions identify barriers and provide suggestions for overcoming them | K8. Provides a list of potential barriers to implementation and solutions to address them | SSE8. Expresses confidence in their ability to identify problems and respond to them during implementation | OE8. Expects that timely identification of problems will facilitate addressing the barrier | NB8. Believes that other champions in other clinics have a role that includes identification of barriers and development of solutions |
