# Supplementary material for: Using intervention mapping to develop an implementation strategy to improve timely uptake of streamlined birth-dose vaccines in the Democratic Republic of the Congo
Source: PLOS Glob Public Health. 2024 Jan 25;4(1):e0002641. doi: 10.1371/journal.pgph.0002641 (PMC10810515; doi:10.1371/journal.pgph.0002641)
Supplement: S2 Table — (DOCX) [file pgph.0002641.s002.docx]

# S2 TABLE. DETERMINANTS, THEORETICAL-BASED METHODS, AND PRACTICAL APPLICATION FOR EACH STUDY ACTOR

| **Determinants** | **Theory-based method** | **Study Actors** | | | | | |
| --- | --- | --- | --- | --- | --- | --- | --- |
|  |  | Implementer | Decision-makers | Vaccine staff | Midwives | CHWs | Champions |
| Knowledge | Technical assistance | Provide ongoing assistance to facilities |  |  |  |  |  |
|  | Organization planning |  | Hold meetings to discuss maintaining the intervention and work flow maintenance |  |  |  | Hold meetings to discuss lessons learned through modeling guideline-based practices |
|  | Perceived severity / instruction | Develop educational material and script for midwives to use in order to disseminate information about vaccines |  |  | Disseminate instruction about severity of diseases to mothers | Disseminate instruction about severity of diseases to community |  |
|  | Information delivery and processing | Face to face training held over two days with champions  Develop and share evidence-based protocol, training materials, educational materials, and facility guidelines |  |  |  |  | Conduct training with relevant facility staff |
|  | Discussion |  |  | Discuss problems encountered collaborating for newborn vaccination or inputting vaccine data with champions | Discuss problem encountered during ANC to delivery visits with champions | Discuss problems encountered when conducting outreach visits with champions |  |
| Skills & Self-efficacy | Skill building & guided practice | Administer skills training in how to use guidelines and incorporate active learning during champion training modeling intervention scenarios |  | Role play to practice conducting intervention during training | Role play to practice conducting intervention during training | Role play to practice conducting intervention during training | Administer skills training in how to use guidelines and incorporate active learning during staff training |
|  | Reinforcement |  | Reinforce the use of the new facility guidelines and intervention materials |  |  |  | Reinforce the use of the new facility guidelines and intervention materials |
|  | Monitoring and feedback |  |  |  |  |  | Regularly monitor and provide feedback to facility staff about skills observed |
|  | Environmental restructuring | Alter vaccine fees to be low (free) and consistent across facilities | Integrate guidelines and materials into a workflow that prompt facility staff to apply guidelines to every mother-infant pair | Facility staff will round together to review new guidelines and protocols with champions   Provide transportation vouchers for mothers who used public transportation to attend vaccine appointment | Facility staff will round together to review new guidelines and protocols with champions | Facility staff will round together to review new guidelines and protocols with champions |  |
| Outcome expectations & Normative beliefs | Persuasion | Invite facility decision-makers to participate in intervention   Train champions in the benefit of guideline-based practice | Select one champion from each of the three facility staff levels (vaccines, midwives, CHWs) to act as champion from their department |  | Disseminate vaccine information effectively during ANC clinics |  | Train facility staff in the benefit of guideline-based practice |
|  | Modeling | Provide feedback to champions about regular modeling of guideline-based practice |  |  |  |  | Model the guideline-based practice on an ongoing basis with implementers |
|  | Cultural relevance | Translate training materials, educational materials, and facility guidelines to contextual languages |  |  |  |  |  |
|  | Evaluation | Oversee baseline and endline evaluation of intervention   Use evaluation findings to advocate to policy-makers for HepB-BD introduction and subsidies for vaccines and transportation vouchers | Support process collecting baseline and endline evaluation of intervention | Monitor infant vaccine rates regularly to facilitate evaluation |  |  |  |
| ** Methods derived using Social Cognitive Theory and Theory of Planned Behavior* | | | | | | | |
